# Evaluating disparities in the U.S. technology transfer ecosystem to improve bench to business translation

**DOI:** 10.12688/f1000research.14210.1

**Published:** 2018-03-15

**Authors:** James Weis, Ashvin Bashyam, Gregory J. Ekchian, Kathryn Paisner, Nathan L. Vanderford

**Affiliations:** 1 MIT Biotechnology Group, Massachusetts Institute of Technology, Cambridge, MA, USA; 2 Department of Electrical Engineering & Computer Science , Massachusetts Institute of Technology, Cambridge, MA, USA; 3 Computational & Systems Biology Initiative, Massachusetts Institute of Technology, Cambridge, MA, USA; 4 Computer Science & Artificial Intelligence Laboratory, Massachusetts Institute of Technology, Cambridge, MA, USA; 5 Koch Institute for Integrative Cancer Research, Massachusetts Institute of Technology, Cambridge, MA, USA; 6 Department of Materials Science & Engineering, Massachusetts Institute of Technology, Cambridge, MA, USA; 7 KP2 LLC, Oakland, CA, USA; 8 Markey Cancer Center, University of Kentucky, Lexington, KY, USA; 9 Department of Toxicology and Cancer Biology, College of Medicine, University of Kentucky, Lexington, KY, USA

**Keywords:** Commercialization, Technology Transfer, Technology Licensing, Patents, Licenses, Startups

## Abstract

**Background:** A large number of highly impactful technologies originated from academic research, and the transfer of inventions from academic institutions to private industry is a major driver of economic growth, and a catalyst for further discovery. However, there are significant inefficiencies in academic technology transfer. In this work, we conducted a data-driven assessment of translational activity across United States (U.S.) institutions to better understand how effective universities are in facilitating the transfer of new technologies into the marketplace. From this analysis, we provide recommendations to guide technology transfer policy making at both the university and national level.

**Methods:** Using data from the Association of University Technology Managers U.S. Licensing Activity Survey, we defined a commercialization pipeline that reflects the typical path intellectual property takes; from initial research funding to startup formation and gross income. We use this pipeline to quantify the performance of academic institutions at each step of the process, as well as overall, and identify the top performing institutions via mean reciprocal rank. The corresponding distributions were visualized and disparities quantified using the Gini coefficient.

**Results:** We found significant discrepancies in commercialization activity between institutions; a small number of institutions contribute to the vast majority of total commercialization activity. By examining select top performing institutions, we suggest improvements universities and technology transfer offices could implement to emulate the environment at these high-performing institutions.

**Conclusion: **Significant disparities in technology transfer performance exist in which a select set of institutions produce a majority share of the total technology transfer activity. This disparity points to missed commercialization opportunities, and thus, further investigation into the distribution of technology transfer effectiveness across institutions and studies of policy changes that would improve the effectiveness of the commercialization pipeline is warranted.

## Introduction

The transfer of inventions from academic institutions to private industry is a major driver of economic growth and human welfare. Broadcom, Google, Akamai, Yahoo, Biogen, Bose, and Genentech represent just a handful of pioneering companies with academic roots ( Kenney, 2017). Indeed, many of today’s defining technologies originated in academic labs, including nuclear energy and the internet ( Busbin, 1995; Manyika & Roxburgh, 2011; Nelson & Byers, 2015).

Technology-driven progress demands not only the development of new inventions, but also their dissemination throughout society. Our national capacity to fuel growth and improve human well-being through new technologies depends on our ability to pass these technologies through a commercialization pipeline. This national need for an efficient and effective technology handoff between academia and industry motivated our analysis of the current United States (U.S.) academic technology transfer environment.

Leveraging data from the Association of University Technology Managers (AUTM) U.S. Licensing Activity Survey, we characterized the performance of research organizations across different steps of the technology transfer process. Our findings indicate that the translational abilities of research organizations across the U.S. vary widely, with a small minority of institutions producing the vast majority of technological and economic benefits. To begin addressing this gap, we surveyed initiatives aimed at improving technology transfer and propose remedies for observed disparities in institutional performance.

## Methods

### Defining the commercialization pipeline

**Figure 1.**
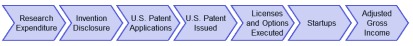
Commercialization pipeline. Each step in this pipeline corresponds to a metric in the AUTM survey. We use the health of the pipeline as a proxy for the overall health of the U.S. technology transfer ecosystem.

The AUTM Licensing Survey solicits responses annually from around 300 institutions, including universities, hospitals and research institutions, to quantify the total technology transfer activity at these institutions. These metrics are derived from a set of core questions that AUTM deems essential for assessing transfer and licensing activity. A detailed description of each metric from the AUTM survey data is given in Supplementary Table 1. We defined the “commercialization pipeline” ( Figure 1) by identifying a set of key questions asked in each AUTM survey, and extracting relevant data from the 2010 to 2014 AUTM surveys. We use this commercialization pipeline to measure and compare relative levels of technology transfer activity at different institutions, and at different steps along the pipeline. The distributions of each metric across every surveyed institution are visualized as linear and log histograms, as well as empirical cumulative distributions, in Supplementary Figure 1 and Supplementary Figure 2.

### Identification of top performing institutions

We ranked each institution from the AUTM Licensing Survey data by each step in the commercialization pipeline. Any institution ranked in the top 10 (about the top 5%) in at least one stage of the pipeline was included in the our list of top performing institutions. This resulting list of 25 institutions (approximately 12% of all surveyed institutions) was then sorted based on mean reciprocal rank (MRR):

$$MRR=\frac{1}{N}\sum_{i}\frac{1}{R_{\mathrm{i,}}}$$

where N = 7 is the number of stages in the pipeline and *R _i_* is the ranking of the institution in step *i* of the pipeline. We chose this scoring system to identify institutions with consistently high performance across the commercialization pipeline while avoiding heavily penalizing anomalous weak performances in just a single metric.

### Calculation of the Gini coefficient

Given value *x _i_* for institution i and *x _j_* for institution j, we calculate G, the Gini Coefficient, such that:

$$G=\frac{\sum_{i=1}^{n}\sum_{j=1}^{n}\left|x_{i}-x_{j}\right|}{2\sum_{i=1}^{n}\sum_{j=1}^{n}x_{j}}=\frac{\sum_{i=1}^{n}\sum_{j=1}^{n}\left|x_{i}-x_{j}\right|}{2n\sum_{i=1}^{n}}.$$

The Gini coefficient is a measure of statistical dispersion used to assess inequality in a population. A high Gini coefficient indicates high levels of inequality where, in this case, a few institutions contribute a substantial amount of total translational activity. Conversely, a low Gini coefficient indicates that each institutions contributes an equal share.

### Statistical analysis

Variance estimates (υ) for the Gini coefficient for each step were derived via jackknife resampling ( Karagiannis & Kovacevic’, 2000; Yitzhaki, 1991):

$$\upsilon =\frac{N-1}{N}\sum_{i=1}^{N}{\left(G_{i}-G\right)}^{2}$$

where *N* is the number of observations, *G* is the Gini coefficient when all observations are considered, and *G _i_* is the Gini coefficient value when the ith observation is removed. The confidence intervals of the log-normal fits were computed to the 95% confidence levels using the Jacobian of the parameter estimates assuming normally distributed residuals. All statistical analysis was performed in MATLAB 2016b (Mathworks, Natick, MA, USA).

## Results

### Inequality between institutions through pipeline

Our goal was to understand how much each institution contributed to each step of the commercialization pipeline and to determine any notable overall trends in U.S. technology transfer. Histograms ( Supplementary Figure 1 and Supplementary Figure 2) of contributions from each institution along the commercialization pipeline reveal highly skewed distributions. The distributions of each metric are generally well approximated by a log-normal fit. Note that the x-axes is on a log scale and therefore the significant skew in the distribution is not immediately apparent. The effectiveness of a log-normal fit decreases towards the end of the commercialization pipeline (Startups and Adjusted Gross Income).

**Figure 2.**
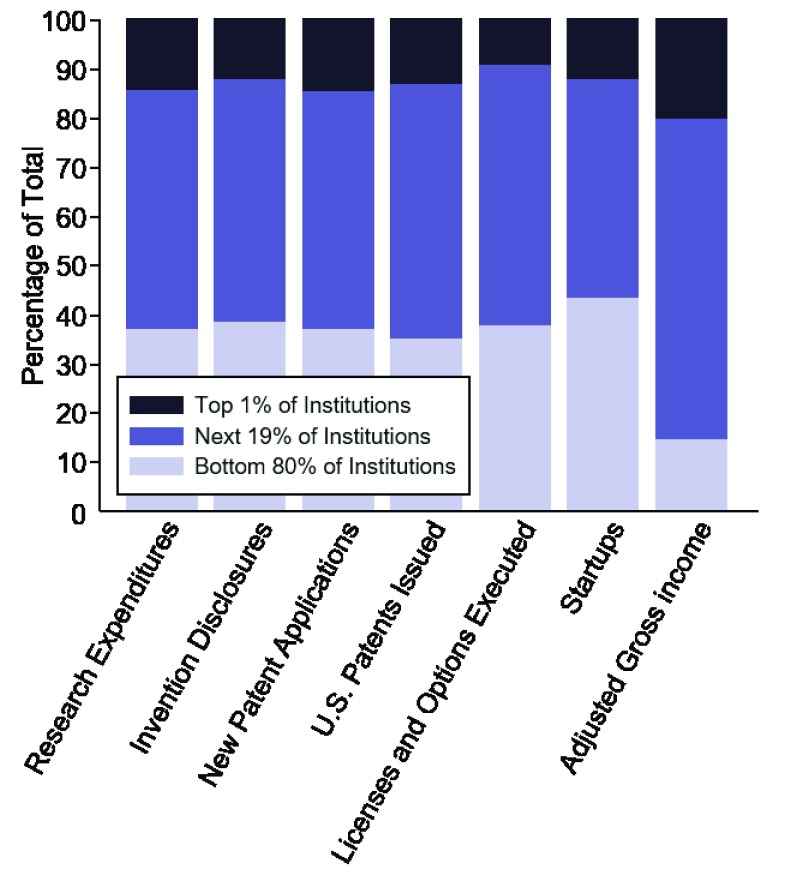
Contribution by top 1%, top 20% and bottom 80% of institution to each step of the commercialization pipeline. A small number of institutions contribute to the majority of commercialization activity.

The majority of institutions contribute a small amount to overall technology transfer regardless of how activity is measured ( Figure 2 and Supplementary Figure 3). Specifically, the top 20% of institutions contribute over 60% of total commercialization activity. Importantly, this trend is robust to normalization by research expenditures, which indicate that differences in research funding do not explain the gap in productivity ( Supplementary Figure 3). In fact, the top 10% of institutions contribute over 40% of “startups per dollar of research expenditures” and over 70% of “adjusted gross income per dollar of research expenditures”.

### Highly performing institutions

We identified the 25 top-performing institutions by sorting all top-performing institutions by the average of their reciprocal ranking at each step in the commercialization pipeline ( Table 1). Most organizations that perform well do so across the entire commercialization pipeline, indicating strong and broad technology transfer abilities (e.g. University of California and University of Texas Systems; MIT; and Stanford). On the other hand, some organizations excel in only specific parts of the commercialization pipeline (e.g. University of Washington in Licenses and Options Executed; California Institute of Technology in New Patent Applications; and University of Georgia in Licenses and Options Executed), which reveals focused, less-robust technology transfer capabilities.

**Table 1.** The 25 top-performing institutions. Bar plots show the mean value over the years under consideration for each institution for each step in our commercialization pipeline.

Institution	Research Expenditures ($M)		Invention Disclosures		New Patent Applications (US)		Patents Issued (US)		Licenses and Options Executed		Startups	
University of California System	5364	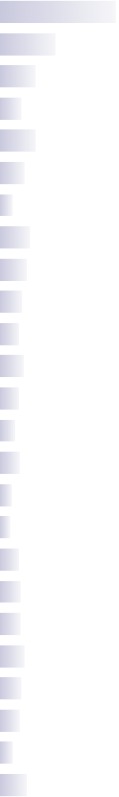	1605	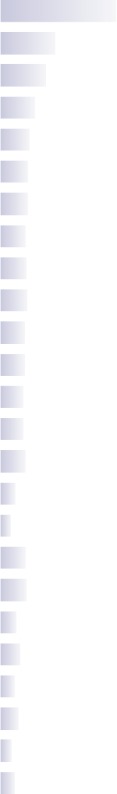	1117	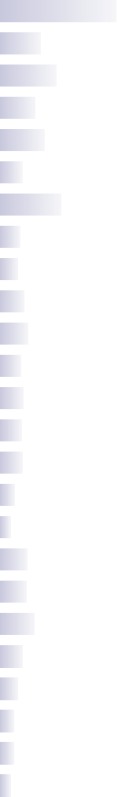	359	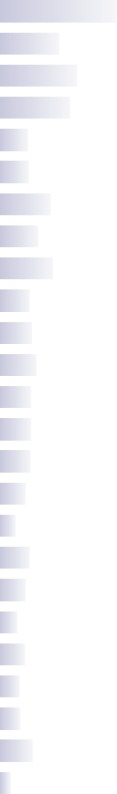	261	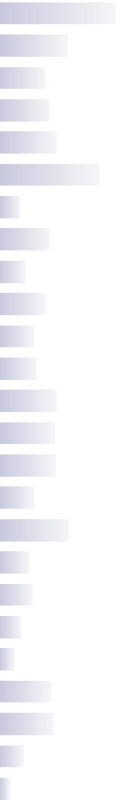	66	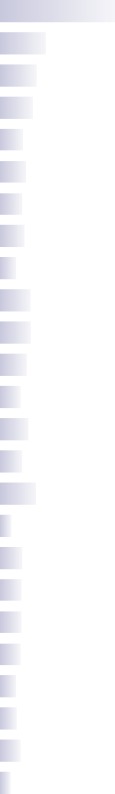
University of Texas System	2508		772		357		173		155		24	
Massachusetts Inst. of Technology (MIT)	1515		646		514		232		107		18	
Stanford University	855		492		308		210		116		16	
Johns Hopkins University	1540		417		400		72		132		10	
University of Washington/Wash. Res. Foundation	1010		401		172		75		225		12	
California Institute of Technology	426		389		565		149		51		10	
University of Michigan	1257		363		152		106		115		11	
UW-Madison/WARF	1113		378		129		153		63		6	
University of Pennsylvania	886		386		196		78		107		15	
Columbia University	737		356		230		86		82		15	
University of Illinois, Chicago, Urbana	972		354		159		102		87		12	
Massachusetts General Hospital	744		326		185		83		132		9	
University of Florida	548		330		167		84		128		14	
Cornell University	781		363		175		82		130		10	
University of Utah	401		223		99		67		82		18	
University of Georgia	307		163		56		34		157		3	
Georgia Institute of Technology	741		364		228		79		72		10	
Harvard University	812		377		213		66		78		9	
University of Colorado	809		239		297		39		53		9	
University System of Maryland	998		292		180		64		38		9	
Duke University	845		212		127		47		118		6	
University of Pittsburgh	755		264		89		49		124		6	
University of South Florida	441		177		89		89		58		9	
Johns Hopkins University Applied Physics Laboratory	1101		219		59		18		29		3	

### Dispersion analysis

We extended this analysis by calculating the Gini coefficient, a measure of statistical dispersion that is often used to quantify income inequality ( Gini, 1912). In this analysis, a low Gini coefficient indicates that each institution is contributing roughly equally to U.S. technology commercialization, whereas a high Gini coefficient indicates that a few institutions are producing the majority of the commercialization output.

**Figure 3.**
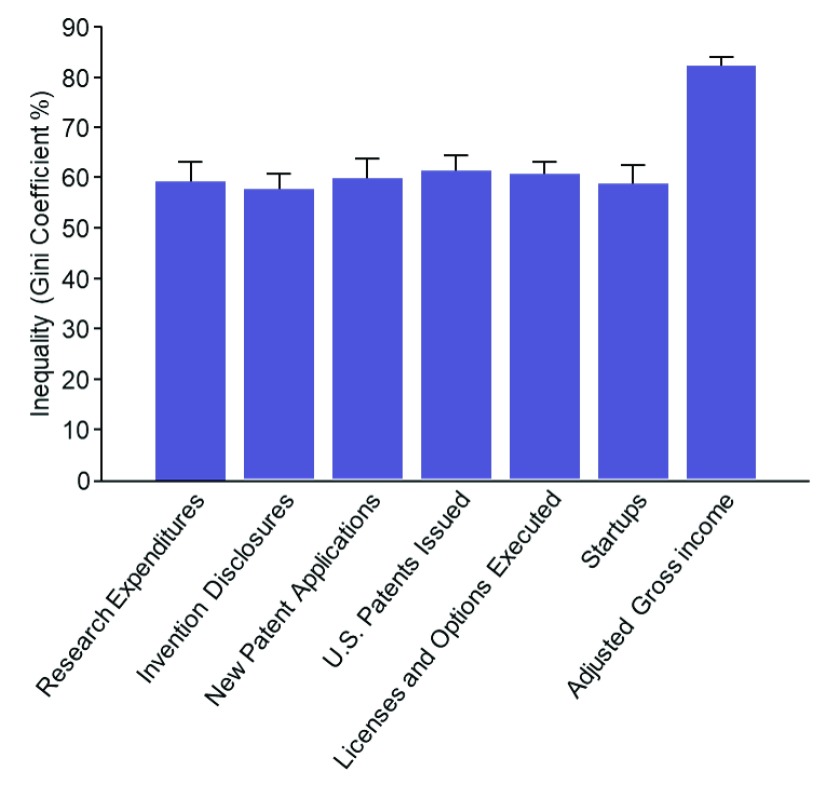
The Gini Coefficient for each stage in the commercialization pipeline, with G of 0% representing complete equality and G of 100% represents complete inequality. Error bars represent one standard deviation of uncertainty as estimated via jackknife resampling ( Karagiannis & Kovacevic’, 2000; Yitzhaki, 1991).

As shown in Figure 3 and Supplementary Figure 3, high levels of inequality exist throughout the pipeline. For context, the Gini coefficient of patents issued in the U.S. is above 60%, while the Gini coefficient of all U.S. household income is 48% ( U.S. Census Bureau). We believe this indicates that the majority of U.S. research organizations have significant untapped commercialization potential, the full realization of which could lead to new technologies and, overall, improved U.S. productivity.

### Improving the pipeline

Many of the top performing institutions have invested significant effort and resources in supporting entrepreneurs at each stage of the commercialization pipeline. Top performing institutions have ensured continuity in their support structure to enable the efficient and effective translation and development of both institute-owned and student-created intellectual property. Table 2 highlights active programs at MIT and Harvard, two top performing translational institutions. Our summary of these initiatives span university incubators, student organizations, university venture capital funds and business plan competitions ( Table 2).

**Table 2.** Current programs at MIT and Harvard, two of the top-performing institutions, that strengthen the commercialization pipeline. The shaded regions denote which areas of the pipeline each program most directly addresses.

	Research Expenditures	Invention Disclosures	New Patent Apps (U.S.)	Patents Issued (U.S.)	Licenses and Option Executed	Startups	Adjusted Gross Income
**Harvard**			**Harvard Life Labs** A 15,000-square-foot shared laboratory space for high-potential life sciences and biotech startups founded by Harvard				
			**President’s Innovation Challenge** A Campus-wide competition supporting student ventures through networking events, mentorship and funding				
	**Harvard Catalyst Program** An NIH funded center fostering a translational environment enabling collaboration and providing tools, training and technologies to investigators						
			**Harvard Biotechnology Club** A student organization that hosts events and provides educational services that allow members to explore the world of business and biotechnology				
**MIT**			**The Engine** An institute-backed venture capital fund empowering disruptive technologies with the long-term capital, knowledge, and specialized equipment and labs they need to thrive				
			**$100K Entrepreneurship Competition** A student run entrepreneurship contest offering mentorship from venture capitalists, serial entrepreneurs, corporate executives, and attorneys, media exposure, prototyping funds, business plan feedback, and discounted services				
			**Sloan Healthcare Innovation Prize** A student run pitch competition supporting early-stage healthcare startups with feedback from industry professionals, pitch workshops, and funding				
	**Sandbox** An institute-backed seed funding for student-initiated entrepreneurship ideas, mentoring from both within and outside of MIT, and tailored educational experiences						
			**Deshpande Center for Technological Innovation** Support for bringing early-stage technologies to the marketplace in the form of breakthrough products and new companies through grants, mentorship, industry connections, and an annual symposium				
			**MIT Biotechnology Group** A student organization that buildings strong, symbiotic relationships between the MIT community, academia, and industry and serves the MIT community by facilitating development of knowledge, skills, networks, and experiences to prepare members for biotechnology-related careers				
					**MIT Life Sciences Alumni Angels** An alumni run angel investing network that supports MIT startups focused on the life sciences with funding, connections, and mentorship		

The overview of successful programs ( Table 2) provides a blueprint for universities that would like to foster improved technology transfer and innovation. While some of these programs would require a significant undertaking on the part of the university, many can be achieved in a straightforward and lightweight manner via the support of student-led activities and partnership with government and private organizations. Examples of grassroot student groups that have launched many new programs exist at both MIT and Harvard. For instance, the MIT Biotech Group group has partnered with the MIT Alumni Angels of Boston to launch a life sciences-focused track to improve access to capital for early-stage startups. The Harvard Biotechnology Club runs an incubator program to develop and translate academic research. These programs represent student-led efforts that require little to no university expenditure or resources. For larger undertakings, university/corporate collaborations can provide an efficient means to achieve significant progress. A prime example of this is JLABS @ M2D2, the medical device incubator partnership between Johnson & Johnson and the University of Massachusetts Lowell ( McCarthy *et al.*, 2013).

## Discussion

Expense, time, infrastructure, and the lack of partnerships are among the most common barriers to research commercialization and alleviating these bottlenecks allows more inventions to enter the marketplace ( Vanderford *et al.*, 2013). Programs to increase support for inventors at less well performing institutions to file disclosures, pursue patent prosecution, and seek licensing deals could significantly boost translational output. Sharing best practices from the leaders in technology commercialization may help bring more new technologies to market.

### Supporting the commercialization pipeline

One salient feature of the top-performing institutions is their broad portfolio of commercialization-focused initiatives. Individually, these projects typically target only a few steps on our commercialization pipeline (for example, business plan competitions target the latter stages of the technology transfer process). However, the best performing universities have a large number of these efforts which, in aggregate, fully span the commercialization pipeline. This observation indicates a potential strategy for improvement of those less well served technology transfer pipelines; specifically, the cultivation of commercialization focused initiatives, such as incubators, business plan competitions, innovation prizes, law clinics, and student organizations. The value of these efforts goes beyond their immediate impact. For example, although when taken at face value, a business plan competition may seem to serve only the winning team, its merit truly stems from bringing together students, entrepreneurs, investors, and the media in a constructive setting. The resources required for such projects are small, and, given the disparity in commercialization, potential societal benefits are vast.

### Outsourcing technology transfer to a third-party

A clear barrier to effective commercialization of university technology is the widespread lack of access to experienced, motivated, and well-resourced technology transfer offices (TTO). Many institutions are unable to support a comprehensive TTO, hampering efforts to introduce new technology into industry. The use of consultants can help alleviate some shortcomings, but faces its own barriers to widespread adoption ( AUTM Technology Transfer Practice Manual).

Alternatively, a coalition of institutions could create a third-party technology licensing organization whose charter is to serve the technology transfer needs of those institutions. Like a sports agent, this third-party organization would use its expertise to strike technology transfer deals between institutions and licensees, freeing universities to focus on their strengths. Funded directly by the institutions and, in part, by licensing revenue, this organization would have the necessary resources and freedom to hire top-tier technology transfer professionals who can effectively interface between stakeholders in industry and in academia, while negotiating on behalf of the parent institutions. These teams would work to creatively package and license technologies to maximize their utility to society, as well as to assure that the parent institutions receive a fair return on their investment.

Operating outside of the university, this organization would be free to make decisions much more quickly than traditional TTOs. Similarly, its employees would be incentivized to work in the best interest of the parent institutions by ensuring the process is both efficient and maximizes value for all stakeholders. This outsourced model of technology transfer speaks towards the latent need for more efficient, properly incentivized, and more widespread efforts to commercialize academic research and development efforts.

## Conclusion

As the U.S. economy becomes increasingly driven by technological change, understanding and improving the commercialization pipeline is critically important. The significant disparity in technology transfer performance is evident as the top few institutions produce a very large share of the country’s total technology transfer. We believe this disparity points to missed commercialization opportunities, which we as a society are paying for by missing out on potentially highly impactful innovations.

## Data availability

The AUTM Licensing Activity Survey data are available on the organization’s website ( https://www.autm.net/resources-surveys/research-reports-databases/licensing-surveys/) by fee or institutional subscription/membership. As such, the raw data analyzed for this study cannot be provided in the context of this article. The 2010–2014 survey data used for this study was obtained as part of an institutional membership (University of Kentucky).

## Supplementary material

**Supplementary Figure 1.** Histograms of each step in the commercialization pipeline shown in Figure 1. Insets show cumulative distributions, with shaded rectangles indicating the number of institutions necessary to reach 80% of total activity.

**Supplementary Figure 2.** Histograms of each step in the commercialization pipeline shown in Figure 1 with a log x-axis. Note that the x-axes is on a log scale and therefore the significant skew in the distribution is not immediately apparent.

**Supplementary Figure 3.** Quantized and normalized distribution for each step in the commercialization pipeline. The shaded bars represent the percentage of the total of each category owned by institutions in percentiles indicated.

**Supplementary Table 1.** Pipeline descriptions.
