# Supplementary figures and images for: Evaluating disparities in the U.S. technology transfer ecosystem to improve bench to business translation
Source: F1000Res. 2018 Mar 15;7:329. [Version 1] doi: 10.12688/f1000research.14210.1 (PMC5897786; doi:10.12688/f1000research.14210.1)

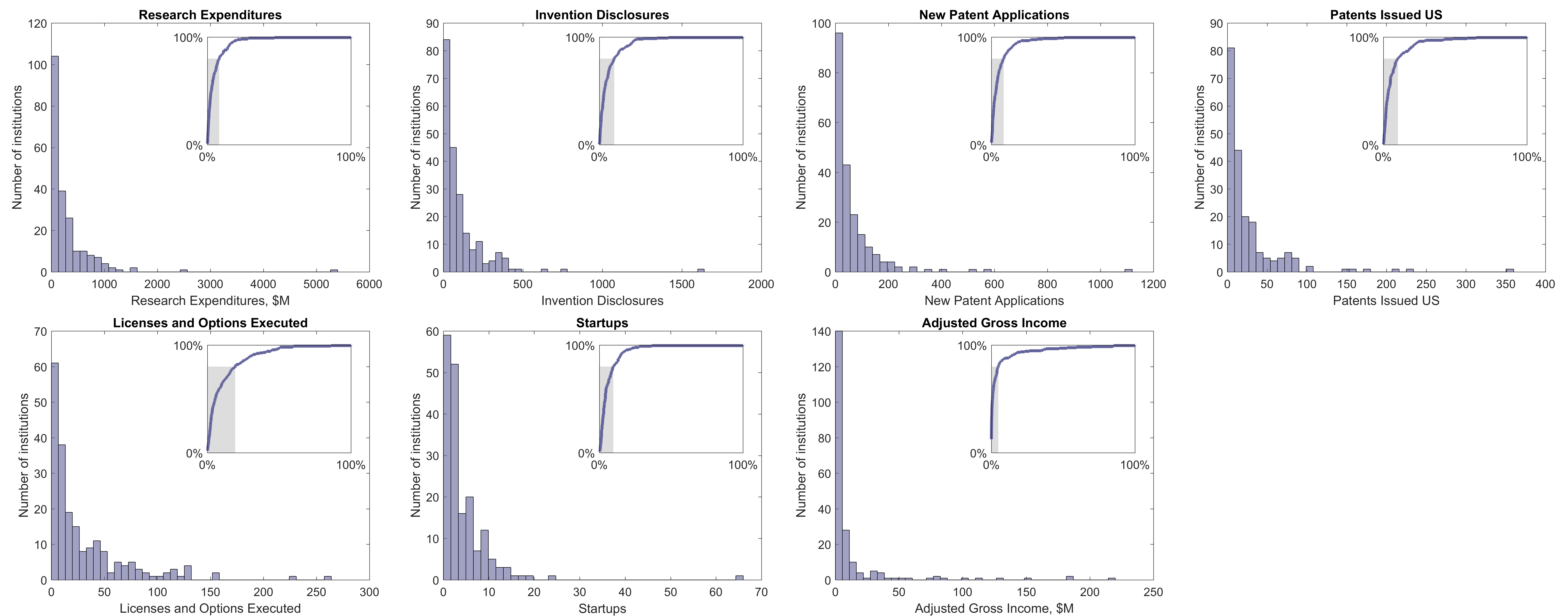

Supplement: Supplementary file 1 [file f1000research-7-15458-s0000.tgz › 5706295f-2564-4b5e-864b-9525ffd5d1b8.png]

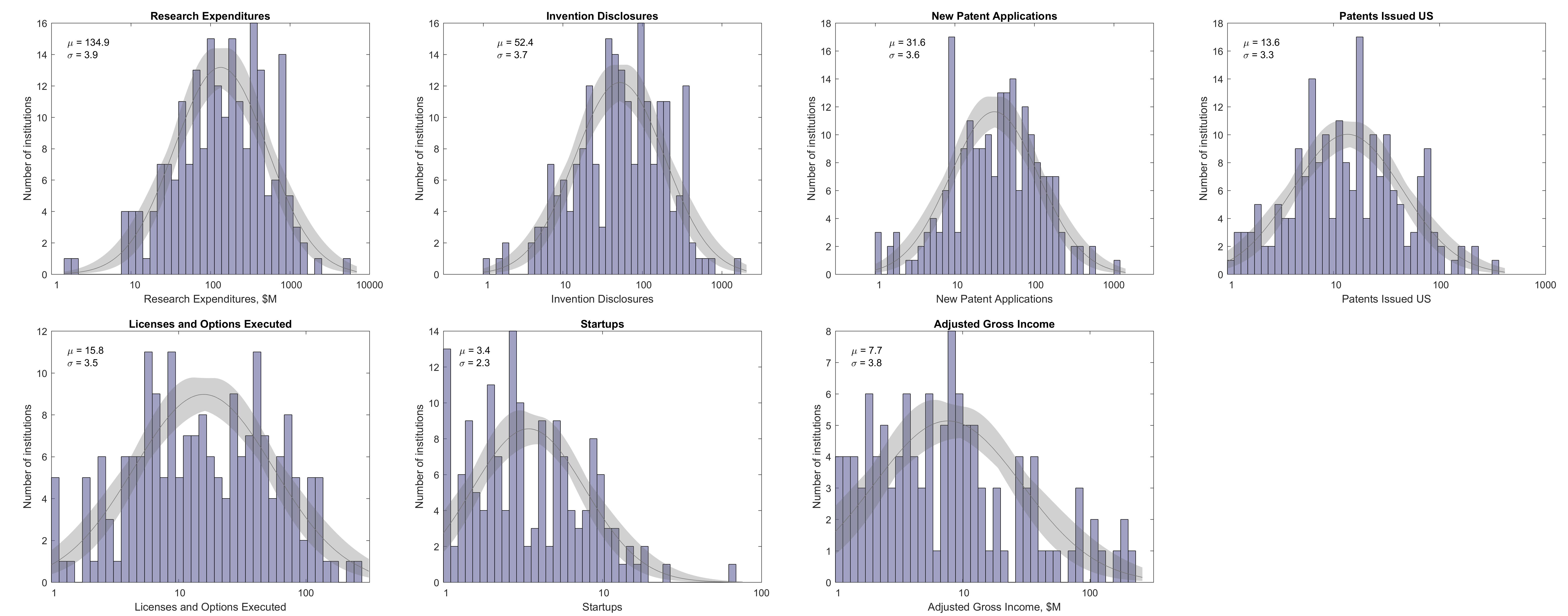

Supplement: Supplementary file 2 [file f1000research-7-15458-s0001.tgz › 8e2ab073-deb4-46c0-a482-db5750635bf5.png]

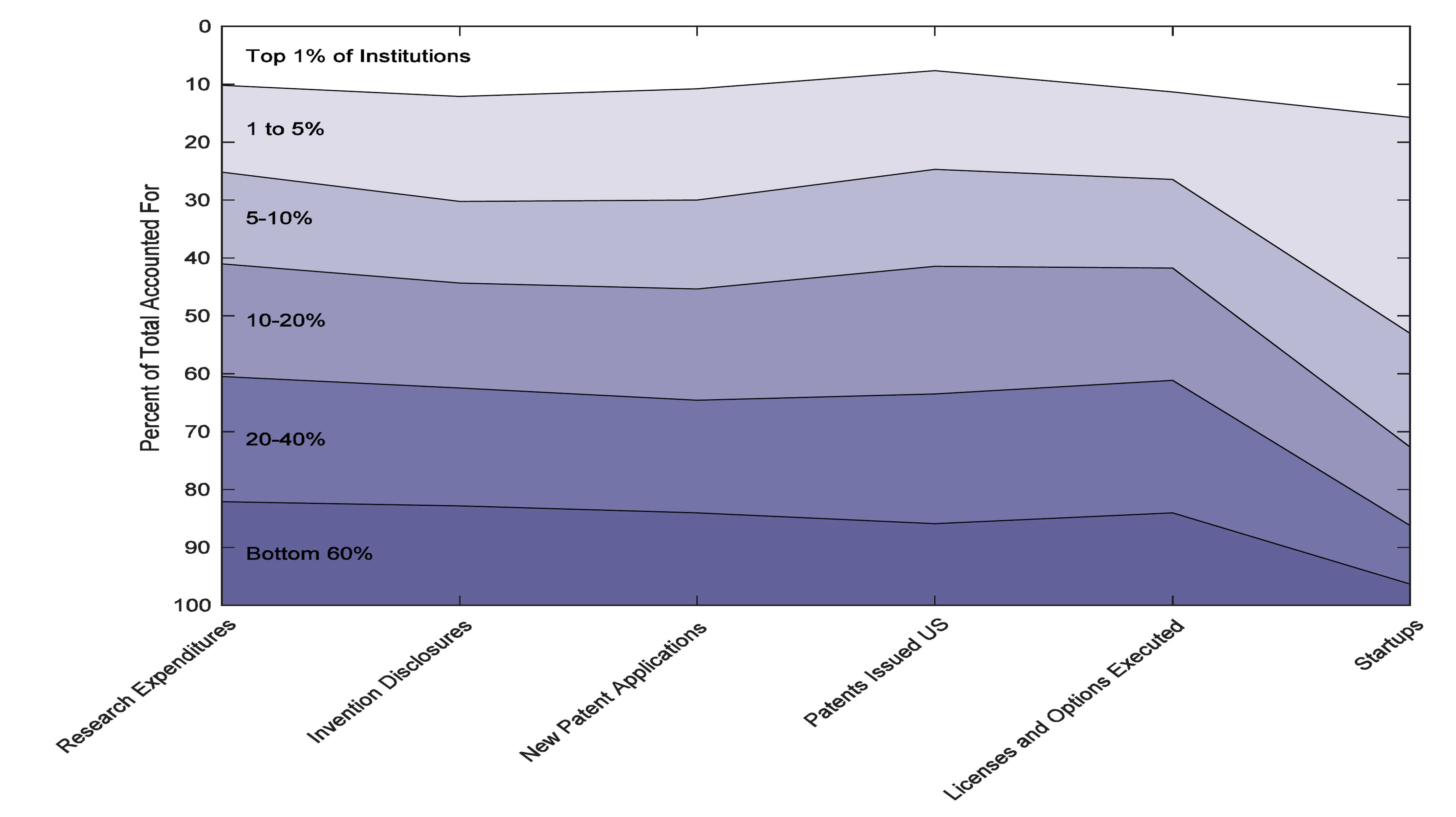

Supplement: Supplementary file 3 [file f1000research-7-15458-s0002.tgz › dfbcaea2-fca7-4e08-b749-3f6c7f83cec8.tif]
