# Supplementary material for: Evaluating disparities in the U.S. technology transfer ecosystem to improve bench to business translation
Source: F1000Res. 2018 Mar 15;7:329. [Version 1] doi: 10.12688/f1000research.14210.1 (PMC5897786; doi:10.12688/f1000research.14210.1)
Supplement: Peer review history

# Peer review: Referee response for version 1

**DOI:** 10.5256/f1000research.15458.r32326

**Published:** 2018-04-11

**Authors:** Dipanjan Nag, Christopher Paschall

**Affiliations:** 1 The Ohio State University, Columbus, OH, USA

Referee #1

- The authors correctly point out that this is a skewed industry, with the top performing schools in any given category contributing more than their share of returns. (Page 1)
- The selection of top 10 institutions in the initial analysis and then expanding to top 25 institutions is somewhat arbitrary. (Page 3)
- This data is coming from institutions of all sizes that vary according to the research dollars as well as the overall size of institutions. For an objective analysis the data needed to be normalized along those lines. (Page 3)
- Some of the stages described by the authors in Figure 1 are metrics that can be manipulated. For example the number of patents applied for and issued really are not metrics of measuring efficiency or impact of an office. Large number of patents can be applied for and granted, the real measure of performance will be the number of patents that are licensed. (Page 3)
- The approach of creating a composite metric is a good one, rather than just looking at the revenue number. The Milken study is another one that has utilized this approach and is an objective measure. The difference is that the Milken study normalized the data, as well as provided the weighting of each factor. (Page 3)
- In most cases the large revenue numbers can be attributed to a single large license or a monetization activity from a university. The weighting of that single license will skew the result enormously. Combined with the lack of data normalization, the results will not be reflective of the true impact to technology commercialization from universities. The weighting factor for each of the "steps" and how they overall impact the ranking is unclear. The weighting of the input metrics vs. the output metrics cannot be the same, hence the rankings would change quite radically. That explanation should be very clearly pointed out. (Page 4)
- The majority of institutions here are based on their size and hence a normalization is needed to avoid picking institutions by size only. Smaller schools often times are more efficient.
- In looking at Table 1 their analysis shows the 1% and the top 19% percent of the institutions. There are 300 institutions in the survey. Why did they pick 25 to rank? 
  In addition Table 1 shows that the top 20% account for 60% of results. Why didn't they cite the top 60 institutions. It would seem logical to look at the top 20%. (Table 1)
- The sharing of best practices has existed for decades in the very collaborative technology transfer community. The AUTM Leadership Conference is precisely that effort, along with the LES IP100 meeting. The fact is that, it is not easy to solicit inventions from faculty. It takes years if not decades to get them on board with the idea that commercialization is a good thing. (Page 6)
- These programs are ideal in nurturing an "ecosystem" for technology commercializatin. The authors are absolutely right about taking a holistic approach rather than just trying to increase disclosures, patents, startups or revenues. (Page 6)
- The whole idea of hiring consultants to fix technology transfer does not work. I have personally been a consultant and a technology transfer professional for many years. The notion of fixing tech transfer through consultant is not economically viable. Consultants most of the time do not have the necessary understanding of the university structure and functions. Many aspects of technology transfer will not have economic impact or metrics based impact, but they need to be done nonetheless. Analogy of hiring consultant would be like replacing full time and dedicated servicemen with mercenaries. Having said that, hiring of consultants for focused engagements is highly recommended and successful offices do that often times in areas such as patent prosecution, valuation, licensing comparable, or market analysis. If there are significant examples that the authors can cite to support the replacement of technology transfer offices with consultants, that would cause readers to consider such an approach. Short of that authors should either reconsider adding this to the article or reframing the recommendation. (Page 6)
- This model has been tried in Japan, where both TTOs and TLOs existed. The model failed miserably. Not only is this model not economically viable, but it creates an atmosphere of trying to create a consortium out of disparate companies like Amazon, Google, Ford and IBM. Some consortiums on a limited scope such as a particular technology focus, e.g. IoT, with a few institutions could work. Again, the authors should cite specific examples that clearly demonstrate the validity of the proposed model otherwise reconsider adding this as a recommendation. (Page 6)
- The picking of two institutions which are ranked according to the authors at #3 and #19 does seem highly biased analysis. Additionally, the programs that the authors have described are student and faculty programs. Technology transfer performed by universities across the board and the data reported by AUTM do not take into account any student programs. Hence, the impact that authors have contemplated at each stage are possibly not accurate. Suggest the inclusion of universities from different "ecosystems" as well as exclusion of student programs from the analysis. (Page 7)
- From a practical standpoint this model will be at best difficult if not impossible with the interest of the parties diverging because of a simple reason, royalty stacking. Each inventor or assignee wants to have the highest value for their portion of the invention. That is just one factor from an economic standpoint. There are many other intangible factors that would be barriers. Anyone who has setup consortia can attest to these challenges. (Page 7)
- Overall, the article has significant challenges:
  1. Authors are predominantly from the Boston area and are analyzing their own institution and another institution which is in close proximity. There are more than 300 institutions and a number of thriving "ecosystems". This is a significant bias and in some ways looks more like a marketing piece as opposed to an objective article. The reason the two institutions are successful could be because of the existence of industry players, local VCs, selective admission of students at two private colleges, and many other factors. 
  2. Their analysis seems to have consisted solely of sorting the AUTM data, not much analysis (interviews, or other supporting data) about the drivers of those outcomes. Hence, they don’t show any support for their statements about technology transfer offices being under performing.
  3. The recommendations or solutions that the authors are proposing are not supported by any data or examples and are simply anecdotal. If such models have existed and worked then analysis of those models would be helpful. 
  4. As we all know the Milken study was published with different results than the authors present. They do not reference the Milken study or explain the differences. Other articles which have attempted to perform similar analysis have cited the Milken study. (e.g. Mature Biotech Entrepreneur in 2014)
  5. Technology transfer is indeed a complex interplay of a various factors that are not always easy to understand. The intangible factors such as location, concentration of research areas, public versus private institution, can all have impact that cause significant disparity in the results. Authors need to consider analyzing or at least mentioning some of those factors in this article. (Page 8 - Conclusion)

Referee #2

1. The years 2010-2014 is a small, five-year sample of data, from which broader trends and conclusions are difficult to draw. The industry moves and changes rapidly, so the 2014 data (which is FY2014, or July 2013 – June 2014 for most institutions), is a relatively old data set.
2. Why were only the top 10 in each category of the Commercialization pipeline included in the MRR analysis?
3. The parameters identified in the Commercialization pipeline (Figure 1) to measure the health of a tech transfer office are, in many instances, inadequate or irrelevant. If one is trying to measure the health of a *university* (not simply its TTO), then one might use research expenditures as a reasonable variable. However, this reviewer fails to see the direct correlation between an institution’s research expenditures and health of the TTO. Furthermore, technology commercialization is a non-linear, dynamic process. The notion that commercialization proceeds from left-to-right (i.e. research expenditure-invention-patent-license-startup-revenue) is incomplete.
4. Similarly, with respect to patent applications and patents issued, such numbers depend heavily on an individual institution’s IP strategy. For example, California Institute of Technology, files provisional patent applications on nearly every invention disclosure that is received. How does such a policy speak to the health of the TTO?
5. Licenses and options are certainly one reasonable metric that can be used to assess the health of a TTO. However, all licenses and options are not created equal. Some universities will license 30 technologies or patents in a single license, and some will license the same 30 technologies or patents in 30 different licenses. Is the second university healthier than the first? The authors have failed to address or account for differences in quality of licenses and options between universities. The quantities by themselves don’t paint an accurate picture.
6. For the Startups metric, the authors should address, again, the quality of the startups coming out of the universities. Some universities create large numbers of startups in a given year, but some of those may not be legitimate, growth-oriented startups with qualified management.
7. Adjusted Gross Income should not be used as a metric to measure the health of TTOs. The TTO has no control over this value. 
8. In the Results section on page 4, I find it disappointing that the authors go through so much analysis to demonstrate the truth of the Pareto principle in technology transfer. The fact that 20% of the universities contribute to 60% of “total commercialization activity” is entirely unsurprising.
9. Again, in the Results section on page 4, “Highly performing institutions”, I don’t see that the data were normalized for research expenditures. If so, the analysis needs to be rerun. Furthermore, the authors need to remove the University of California, University of Texas, and University of Maryland systems because those systems report to AUTM statewide. Alternatively, the authors could retrieve any desired data from the individual institutions in those states.
10. I am not clear on the analogy between the Gini coefficient of patents and US household income. How do the authors conclude that a 60% Gini coefficient for patents means that universities have significant untapped commercialization potential?
11. Page 5, “Improving the pipeline”, along with Table 2, reads more like an advertisement for Harvard and MIT, particularly the Biotechnology Clubs of which some of the authors are members. The paper would be strengthened if they identified activities conducted outside of Boston-based universities that are contributing unexpected positive results to the tech transfer ecosystem.

 

 Discussion section comments

1. Sharing best practices across institutions is a common and frequent activity in the university tech transfer community. Organizations like AUTM exist for such a reason.
2. Where do the authors propose universities obtain resources to promote additional commercialization activity? 
3. Do the authors have any data to support one of their proposed solutions, the outsourcing of technology transfer to consultants?

 

 Overall questions and comments

1. Specifically, outside of Harvard and MIT, what are institutions in the top 25 doing that other institutions can replicate to improve their TTOs? 
2. Are the authors aware of similar programs at other universities as those highlighted in Table 2?
3. Have the authors considered non-traditional factors that may contribute to the success of TTOs, such as overall university mission, geographic location, appetite for risk, patience for technology development timelines, etc.?

We have read this submission. We believe that we have an appropriate level of expertise to confirm that it is of an acceptable scientific standard, however we have significant reservations, as outlined above.

## References

[1] WeisJBashyamAEkchianGPaisnerKVanderfordN: Evaluating disparities in the U.S. technology transfer ecosystem to improve bench to business translation. *F1000Research*.2018;7: 10.12688/f1000research.14210.1 10.12688/f1000research.14210.1 PMC589778629721313

# Peer review: Referee response for version 1

**DOI:** 10.5256/f1000research.15458.r32093

**Published:** 2018-04-09

**Authors:** Evan Facher

**Affiliations:** 1 Innovation Institute, University of Pittsburgh, Pittsburgh, PA, USA

The authors tackle a very relevant question virtually all technology commercialization organizations at academic institutes have – what can they do to enhance translation of innovations developed at their organizations. In order to assess the current situation and provide some thoughts on approaches to ameliorate the challenges their colleague institutes have, the authors took a well reasoned, data intensive approach to examine a large set of information. The AUTM data is a valuable source of self-reported figures and a large amount of it was assessed to reach the presented conclusions. The design was appropriate and relevant to answer the hypotheses posed in this article. Further, a fairly robust statistical analysis was performed. Given the data is available (for purchased access) to any institute of higher learning, the analyses described in the paper can be readily replicated, and potentially expanded on, by any group that seeks to build on it.

 The conclusions drawn as a result of this assessment are supported by the data and provide multiple areas for practioners of technology transfer to examine further for implementation. I believe the manner in which the authors broke down the commercialization pipeline was sound and is a good model for others to use in assessing their own processes. The only suggestion I would offer is that universities are increasingly focusing on software licensing, as a result, not all translation will be of patented innovations but rather copyrights. As such, there may be additional information that can be garnered for these inventions. One additional area that may be interesting to include for the authors if they chose to eventually expand on the analysis beyond this submission is understanding the disparity of human resources at the AUTM schools to understand how much, if any, this specific infrastructure component is tied to commercialization activity and AUTM metrics.

 In conclusion, I enjoyed the opportunity to review this article and believe it adds to the literature on technology transfer. A significant amount of data was used to identify recommendations that align with opportunities most organizations can adopt.

I have read this submission. I believe that I have an appropriate level of expertise to confirm that it is of an acceptable scientific standard.

# Peer review: Referee response for version 1

**DOI:** 10.5256/f1000research.15458.r32556

**Published:** 2018-04-06

**Authors:** Adriana Bankston

**Affiliations:** 1 Future of Research, Abington, MA, USA

**General comments:**

 This publication highlights an important topic in examining the effectiveness of universities to facilitate the transfer of new technologies into the marketplace. This work also raises awareness to the fact that only a small number of institutions contribute to the vast majority of the total commercialization activity. This is only one of the many disparities found across U.S. institutions, and the work presented here very effectively puts this particular disparity into a broader context.

 Therefore, this type of research has the potential to be utilized as a way to press for change in the system as a whole. Particularly for this field, a change in the system would require advocating for the implementation of successful practices from high-performing institutions across a higher number of U.S. institutions, so that eventually these technologies can become commonplace in all universities. To this end, I believe it is particularly important to consider how we might provide similar types of resources to U.S. institutions with less of an access to these tools or with fewer number of tools at their disposal as compared to the high-performing institutions in the area of technology transfer.

 The publication itself is very well-written, with the motivation for pursuing this work and its purpose being very clearly defined. The authors have also very succinctly articulated the gap filled by this work and its overall significance to the field, and the results support the conclusions drawn. Overall, I believe this paper is suitable for publication in its current form. I have just a few suggestions for improvement, or ideas that might be useful to consider if expanding or revising this paper in the future. These are mostly broad recommendations related to whether and how the disparities described here could be reduced across universities.

**General considerations:**

 I would like to commend the authors for detailing the steps of the commercialization pipeline early in the publication - this is very helpful for those who might not be very familiar with it. Similarly, the explanations of the data analysis parameters, including the Gini coefficient, are very useful in understanding the conclusions drawn from these studies. One suggestion I would have is to move the information from the Supplementary Table 1 into a main table in the publication, to enable the reader to more easily and quickly refer to these concepts in conjunction with reading Figure 1.

 I also appreciate the fact that the commercialization pipeline allows the authors to answer key questions using relevant data in the AUTM Licensing Survey. Hower, it would be really helpful to also conduct a more in-depth investigation of how each of the universities in the AUTM Licensing Survey measures up in every step of the commercialization pipeline, in order to obtain a view of the national landscape on this issue beyond just the top 25 institutions. While I realize that looking at all 300 universities may be cumbersome and that analyzing the top 25 was a relevant strategy in this case, a more detailed analysis might allow for looking at broader trends that exist across U.S. institutions in the technology transfer area, which could then be utilized to guide future reforms.

 In terms of the actual reforms, I would also be curious to know more about the types of policies which the authors propose to be implemented in U.S. universities in order to improve the effectiveness of the commercialization pipeline. As part of this question, I recognize the importance mentioning the grassroot student groups who have launched successful programs at MIT and Harvard. Perhaps it would be helpful to suggest how such universities can best share their strategy for success in technology transfer with other universities in the U.S., which could potentially enable the creation of a shared collection of online resources, and/or a network of professionals interested in helping universities improve in this area who would meet regularly to discuss this issue.

 Given that there are multiple programs described from these two institutions in Table 2, it may be helpful to discuss more about the specifics of these programs, in terms of the similarities and differences (perhaps in a supplementary figure) in order to point out particular elements that would be useful for other universities to utilize and incorporate into a more universal program that could be adopted widely across the U.S. to improve the technology transfer field. Alternatively, are there differences between these programs that could be pointed out in order to determine their suitability for being adopted by other universities as multiple independent programs?

 I also wonder if there are other additional types of actions that could be undertaken by graduate students and postdocs who might have an interest in this area, or by university administrators who could assist in potentially reducing some of these disparities described here. One area that might be worth exploring in this regard is implementing technology transfer programs in all U.S. universities (perhaps these could be designed by a local technology transfer office) geared towards training both researchers and administrators in this area, in order to facilitate the commercialization of the findings produced by researchers in U.S. institutions.

 I believe the idea of a third party organization, which is mentioned, could work very well. However, there could be limitations in terms of what universities (or researchers) are willing to share with an outside group, depending on how the agreement terms are formulated. Therefore, it may be useful to also consider other individuals within universities who could be involved in implementing reforms in the technology transfer process from the inside. I also wonder how to ensure that the third party organization will associate with the institutions that are most in need of this partnership, instead of linking to the already high performing institutions, the latter which could in fact ensure their own success. To this end, these agreements would need to be carefully crafted with the interest of both parties in mind.

 At the same time, multiple parties should contribute to the implementation of such reforms, including those outside the university. Perhaps it would be useful to add into the current Figure 1 some suggestions at every step along the way, indicating where improvements could be made and by whom (i.e. which stakeholder). This broader analysis might also help with further discussions of the types of changes which should be implemented in universities in order to ensure that all U.S. institutions have the same resources and as the currently high-performing institutions in technology transfer, therefore aiming to reduce some of these disparities and create a more uniform way to analyze this problem across the board. In addition, as multiple groups are likely to be involved in this process, perhaps it would also be useful to expand this discussion by adding in more details on the efforts already undertaken successfully by each of these groups which could help reduced these disparities. For example this could include direct links to such efforts, with clear instructions and concrete actions which other universities could easily adopt and implement locally.

**Specific suggestions:**

 Overall, the motivation for each part of the study and the actual results are very clearly explained and are insightful. I was particularly intrigued by the findings in Table 1, and the idea that performance of institutions in such these areas can be measured. This is encouraging to see in thinking more broadly about the variables that can be quantified from U.S. institutions. One suggestion is that it may be useful, after each result, or perhaps in the discussion section, to include further explanations of how each of these findings contributes to our broader understanding of academic technology transfer. This might also lead to an obvious list of particular weakness in the commercialization pipeline that must be corrected across institutions.

 Getting more into the data itself, with respect to Table 1, I understand the top performing institutions are ranked as high performing in all of the presented categories. However, there are a few institutions in the list that I would consider high performing academically, but which have been indicated to perform in a variable manner in some of the particular areas examined within the commercialization pipeline. I am wondering whether more discussion on these “gray area” institutions could be added, and how their particular performance in a given area translates to benefiting the economy itself. For example, does high performance in certain variables lead to benefits in the local economy, and is high performance in all of these areas required to benefit the U.S. economy as a whole, or is this a more mixed population?

 Along these lines, in thinking about the differences in available resources for technology transfer between U.S. universities, I also wonder if it is possible to perform a type of analysis that would take into account this difference and measure performance in a way that is dependent upon the available resources. For example, a particular university may be considered successful overall for their local area given the limited amount of resources, depending on how this analysis is performed and analyzed. However, if we only consider universities that are high performing overall in all categories, it may be more difficult to analyze the individual success of those institutions who only show high performance in certain areas.

 Overall, I believe Table 2 contains a wealth of information that could be further analyzed and discussed to draw additional conclusions from the data presented here, including potentially by examining the entire dataset of 300 institutions, which could add another layer of depth and complexity to these findings.

 The authors also mentioned that this analysis was performed using 2010-2014 AUTM Licensing Survey data. As this survey data is being reported every year, it may be interesting in the future to expand beyond this timeframe and examine more general trends in the factors presented here over a longer period of time (for example in the last 10 years). That kind of analysis could also provide more insights into the landscape of technology transfer as a whole, and highlight particular recommendations that could be made (or maybe have already been made) to improve the technology transfer system in the U.S. Finally, if available, obtaining and sharing the raw data from the Survey itself may also allow other individuals to perform their own additional analyses of interest.

I have read this submission. I believe that I have an appropriate level of expertise to confirm that it is of an acceptable scientific standard.

# Peer review: Referee response for version 1

**DOI:** 10.5256/f1000research.15458.r32039

**Published:** 2018-03-19

**Authors:** Karen I. Deak

**Affiliations:** 1 The IDEA Center, University of Notre Dame, Notre Dame, IN, USA

Other than the Materials and Methods dealing with the underpinnings of the statistical methodolgy, which are well beyond me (my failure, not the authors'), the article is clear and easy to understand. It provides an interesting look at the University technology transfer process, and is probably of interest to a wide variety of readers -- both those in the technology transfer field and those who care what federal research dollars can provide.

 The authors demonstrate that some universities are better than others at commercializing the research that their professors undertake. They provide an examination of whether universities can be good at all steps of the process; or whether it is more effective for a TTO to specialize at one, more high-leverage, point in the process. 

 The article is clear, and the conclusions and findings are well-reported. The authors additionally provide a few suggestions of how to improve or make more efficient the technology transfer process. These takeaways will be of interest to readers in TTOs, especially.

 It would be interesting for a follow-on article for the authors to examine tech transfer efforts at different "types" of universities -- with or without a medical school; or at places where there have been blockbuster licensing deals vs. not. But clearly those ideas are well outside the scope of this article.

I have read this submission. I believe that I have an appropriate level of expertise to confirm that it is of an acceptable scientific standard.
